# A Multicentre Hospital Outbreak in Sweden Caused by Introduction of a *vanB2* Transposon into a Stably Maintained pRUM-Plasmid in an *Enterococcus faecium* ST192 Clone

**DOI:** 10.1371/journal.pone.0103274

**Published:** 2014-08-25

**Authors:** Audun Sivertsen, Hanna Billström, Öjar Melefors, Barbro Olsson Liljequist, Karin Tegmark Wisell, Måns Ullberg, Volkan Özenci, Arnfinn Sundsfjord, Kristin Hegstad

**Affiliations:** 1 Research group for Host-Microbe Interactions, Faculty of Health Sciences, University of Tromsø – The Arctic University of Norway, Tromsø, Norway; 2 Unit for antibiotics and infection control, the Public Health Agency of Sweden, Solna, Sweden; 3 Department of Clinical Microbiology, Karolinska University Hospital, Huddinge, Sweden; 4 Norwegian National Advisory Unit on Detection of Antimicrobial Resistance, Department of Microbiology and Infection Control, University Hospital of North-Norway, Tromsø, Norway; University Medical Center Utrecht, Netherlands

## Abstract

The clonal dissemination of VanB-type vancomycin-resistant *Enterococcus faecium* (VREfm) strains in three Swedish hospitals between 2007 and 2011 prompted further analysis to reveal the possible origin and molecular characteristics of the outbreak strain. A representative subset of VREfm isolates (n = 18) and vancomycin-susceptible *E. faecium* (VSEfm, n = 2) reflecting the spread in time and location was approached by an array of methods including: selective whole genome sequencing (WGS; n = 3), multi locus sequence typing (MLST), antimicrobial susceptibility testing, virulence gene profiling, identification of mobile genetic elements conferring glycopeptide resistance and their ability to support glycopeptide resistance transfer. In addition, a single VREfm strain with an unrelated PFGE pattern collected prior to the outbreak was examined by WGS. MLST revealed a predominance of ST192, belonging to a hospital adapted high-risk lineage harbouring several known virulence determinants (n≥10). The VREfm outbreak strain was resistant to ampicillin, gentamicin, ciprofloxacin and vancomycin, and susceptible to teicoplanin. Consistently, a *vanB2*-subtype as part of Tn*1549*/Tn*5382* with a unique genetic signature was identified in the VREfm outbreak strains. Moreover, Southern blot hybridisation analyses of PFGE separated S1 nuclease-restricted total DNAs and filter mating experiments showed that *vanB2-*Tn*1549*/Tn*5382* was located in a 70-kb sized *rep*_17/pRUM_ plasmid readily transferable between *E. faecium*. This plasmid contained an *axe-txe* toxin-antitoxin module associated with stable maintenance. The two clonally related VSEfm harboured a 40 kb *rep*_17/pRUM_ plasmid absent of the 30 kb *vanB2-*Tn*1549*/Tn*5382* gene complex. Otherwise, these two isolates were similar to the VREfm outbreak strain in virulence- and resistance profile. In conclusion, our observations support that the origin of the multicentre outbreak was caused by an introduction of *vanB2-*Tn*1549*/Tn*5382* into a *rep*_17/pRUM_ plasmid harboured in a pre-existing high-risk *E. faecium* ST192 clone. The subsequent dissemination of VREfm to other centres was primarily caused by clonal spread rather than plasmid transfer to pre-existing high-risk clones.

## Introduction

Enterococci, and *Enterococcus faecium* in particular, have undergone a transition from harmless gut commensals to be a leading cause of multidrug resistant hospital infections [1]. *E. faecium* is associated with urinary tract infections, endocarditis, infections in indwelling catheters and septicaemia in hospitalised patients [2], [3]. Notably, a pronounced increase in bacteraemias caused by *E. faecium* in Europe has been reported [4]. The ability of hospital adapted lineages of *E. faecium* to compile antibiotic resistance and virulence factors by horizontal gene transfer might be attributable to this observation [1].

The use of Multi Locus Sequence Typing (MLST) has been considered a standard method for global epidemiological surveillance [5], while pulsed-field gel electrophoresis (PFGE) is the preferred method for examination of *E. faecium* seen in local outbreaks. The clonal complex (CC) 17 has shown to pool hospital associated *E. faecium* strains characterised by a high rate of recombination, multidrug resistance as well as numerous virulence determinants [6]–[8]. Newer insights pertained with another type of population structure analysis, Bayesian Analysis of Population Structure (BAPS) of CC17 strains, show divergent origins of sequence type (ST) lineages within CC17. CC17 strains could largely be divided in two BAPS groups, 2-1 (lineage 78), and 3-3 (lineages 17 and 18), with the corresponding MLST ancestry nodes in parenthesis [9].

A worldwide increased prevalence of acquired resistance to commonly used antibiotics is observed in clinical isolates of *E. faecium*. A total of eight gene clusters, *vanA,B,D,E,G,L,M,N,* have been associated with acquired vancomycin resistance in enterococci (VRE) [10]–[13]. VanA VRE is most prevalent globally, but VanB-type VRE are predominant in Australia and on the rise in many European countries [14]–[19]. The *vanB* gene cluster include three subtypes, *vanB1-3*, conferring inducible low- to high-level resistance to vancomycin and susceptibility to teicoplanin [20]. The predominant subtype *vanB2* is an integral part of an Integrative Conjugative Element transposon family Tn*1549*/Tn*5382* supporting transfer of *vanB2* [21]. Several plasmid replicon types encoding glycopeptide resistance as well as stabilising toxin-antitoxin systems have been linked to CC17 strains [22]. Moreover, a number of putative virulence genes have been associated with *E. faecium* and it is hypothesised that their phenotypes might work in concert to promote host colonisation and subsequent invasion [2].

The prevalence of vancomycin resistant *E. faecium* (VREfm) in Sweden remained low until 2007 when a large hospital associated outbreak occurred [15]. Three hospitals in separate counties were involved. The outbreak was not declared over until 2011.

The aim of the present study was to explore the origin of the outbreak strain by performing molecular characterisation of representative isolates of the outbreak strain and compare them with consecutive invasive *E. faecium* isolates from the same time period and location.

## Materials and Methods

### Bacterial isolates

All cases of vancomycin-resistant enterococci (VRE) are mandatorily reported to the Public Health Agency of Sweden and collected for resistance and epidemiological typing. In 2007, an increasing number of notified VRE-cases were seen in Stockholm County, related to a clonal VanB-type *E. faecium* strain. The strain was subsequently reported in two other geographically distant counties (Västmanland and Halland) [15]. During the autumn 2008, clonally related isolates of VanB-type Efm (n = 17) from separate infection/colonisation events in these three counties were selected for molecular studies. The selected isolates occurred early and late during the outbreak period and represented isolates with divergent resistance profiles and related PFGE subtypes. Four blood isolates from Collection B (see below) were also included in the molecular analyses, yielding a total of 21 isolates in Collection A.

In an attempt to reveal the origin of the *E. faecium vanB* outbreak strain, all consecutive *E. faecium* blood culture isolates from 1^st^ of January 2006 to 31^st^ of August 2009 (n = 191; Collection B) diagnosed at the Karolinska University Hospital Huddinge where the outbreak was first identified, were collected and analysed by PFGE; 2006 (n = 45), 2007 (n = 32), 2008 (n = 71) and 2009 (n = 43). Four of these isolates were selected for further analysis using whole genome sequencing (WGS) with the Roche 454 pyrosequencing platform. The selection criterion was based on PFGE patterns, where the first vancomycin susceptible (VSE1036), the first vancomycin resistant (VRE1044) and the most recent vancomycin resistant (VRE1261) isolate with indistinguishable or closely related PFGE-patterns to the outbreak strain, were chosen. For comparison, one vancomycin resistant isolate (VRE0576) from 2006 was chosen because of its divergent PFGE pattern. *E. faecium* 64/3 [23], BM4105RF and BM4105-Str [24] were used as recipients in filter mating experiments. Isolates from a polyclonal cluster of *vanB2* positive *E. faecium* from 2002–2004 in the Swedish county Örebro [18] were included in the ICE*Slu*van Q8 PCR to evaluate their *vanB* transposon signature.

### Antimicrobial susceptibility testing

The minimum inhibitory concentration (MIC) was determined using Etest (BioMerieux) and interpreted according to the clinical breakpoints of the European committee on Antimicrobial Susceptibility Testing (EUCAST) (www.eucast.org). For ciprofloxacin, a tentative breakpoint was used classifying isolates with MIC >32 mg/L as high level resistant [25].

### PFGE and MLST

For *Sma*I-digestion, the protocol adapted by Saeedi *et al.* [26] was used with 5 U/mL lysozyme added in the lysis buffer. The bands were separated with the following program: Block I switch time 3 to 26,5s for 14 hours and 50 minutes. Block II: switch time 0,5 to 8,5s for 6 hours and 25 minutes. Total run time 21 hours and 15 minutes at 6V with 120°. The PFGE patterns were analysed and compared using BioNumerics software (version 6.6, Applied Maths). The Dice coefficient was used for pair-wise comparison of patterns, and the un-weighted pair group method with arithmetic mean (UPGMA) for pattern grouping. Isolates clustering above 97% were considered identical and isolates with identity >90% closely related.

MLST was performed using the method adapted by Homan *et al.* [5] with the following primers: adk1n, adk2n, atp1n, atp2n, ddl1, ddl2, gdh1, gdh2, gyd1, gyd2, pstS1n, pstS2n, purK1n and purK2n.

### Detection of genes by PCR and isolation of bacterial DNA

Extraction of DNA for all PCRs were performed by BioRobot M48 (Qiagen), according to the manufacturers manual. Primers and positive controls are described in Table S1.

The chosen virulence genes are associated with high-risk genotypes, and included *esp* [27], *hyl* [28], *acm* [29], *efaAfm* [30], *sgrA*, *ecbA*, *scm*, *orf903/2010/2514* and *pilA/B* [31]–[33]. PCRs genotyping presence of *vanB* [20] and linkage to Tn*5382* [34] was done. PCRs were conducted as stated in Table S1. PCR was performed using the JumpStart REDTaq Readymix PCR Reaction mix (Sigma), with a standard program of 1 min in 95°C followed by 30 cycles at 95°C for 30 sec, 30 sec of annealing in the temperature given in Table S1, 72°C for 1 min with a final elongation step at 72°C for 7 min. The presence of *vanB* in transconjugants was tested by PCR using 1 µl of bacterial culture in BHI broth and an additional initial denaturation step of 10 min at 95°C.

### Southern blotting and hybridisation

PFGE analysis of S1-digested DNA was used to analyse the plasmid content. Plugs were made as for *Sma*I digestion, and the digestion was performed as described by Rosvoll *et al.* [22]. The Vacugene XL system (Amersham Biosciences) was used for Southern blotting. Consecutive hybridisation was performed using *rep*_17*/*pRUM_, *vanB* and *axe-txe* probes in the mentioned order. Characterisation of the *rep*_pLG1_ [35] and *rep*_2/pRE25_ [36] plasmid determinants were also done by Southern blotting and hybridisation after S1-nucelase PFGE. Probes were made by amplification using positive controls (see Table S1), and labelled using the PCR DIG synthesis kit (Boehringer Mannheim). The same hybridisation protocol as in Rosvoll *et al.* [22] was used with the following modification: The DNA was purified after the first PCR using the Cycle Pure Kit (zDNA).

### Conjugative transfer of *vanB*

Filter mating was performed according to Bjørkeng *et al.* [18] with some minor modifications, using the *E. faecium* 64/3 and *E. faecium* BM4105-RF as recipient strains for the first filter mating, and BM4015-Str in retransfer. Briefly, the isolates were grown together on MF-Millipore membrane filters for 24 h, spotted on selective BHI agar plates containing either vancomycin (8 mg/L), fusidic acid (10 mg/L) and rifampicin (20 mg/L), or all three antibiotics together. The bacterial suspension was serially diluted down to 10^−9^ and incubated at 37°C for 48 h. In the retransfer experiments, the recipients were selected on plates containing 1000 mg/L streptomycin.

### Whole genome sequencing and analysis

Chromosomal DNA from the four isolates (VSE1036, VRE1044, VRE1261 and VRE0576) was prepared using the DNeasy Blood and Tissue Kit (Qiagen) with lysozyme (20 mg/mL) added to the lysis buffer and further treated with RNase. The protocol also allowed purification of plasmids. Libraries were prepared and used for whole genome shotgun sequencing on a 4-region picotiter plate with the Roche 454 FLX system according to standard protocols (www.454.com). Raw sequencing data were processed with standard filters using the GS Run Processor (v 2.6), generating between 246084 and 310421 reads for each of the strains with average lengths between approximately 307 and 320 nucleotides, corresponding to between 78750175 and 99124035 nucleotides. Reads were assembled *de novo* with the accompanying GS *de novo* assembler software (v 2.6) (454 Newbler algorithm) generating between 201 and 302 contigs with a length of more than 100 nucleotides. The GS Reference mapper software (v 2.6) was subsequently used for homology comparisons between the different strains and to find homologies to specific query gene sequences and also used to identify indels and point mutations that separated the different strains. Some small plasmids could be identified by screening for contigs where individual reads mapped to both ends of the contig. The contigs were tentatively linked to each other by comparison of molecular biology data, identification of individual reads mapping to two different contigs and comparisons with published genomes. The tentative gene content of the contigs was automatically analysed at NCBI with the Prokaryotic Genome Annotation Pipeline (PGAP).

In Figure S1 alignment of genome sequences against the reference genome of Aus0004 (NC_017022) was done by reordering the contigs of VRE1044 against the Aus0004 reference genome using Mauve 2.3.1. The reordered contigs of VRE1044 were then used as reference to reorder the contigs of the other isolates before the genomes were aligned using progressiveMauve [37].

The Whole Genome Shotgun projects have been deposited at DDBJ/EMBL/GenBank under the accessions JAAJ00000000 (VSE1036), JAAK00000000 (VRE0576), JAAL00000000 (VRE1044) and JAAM00000000 (VRE1261). The versions described in this paper are versions JAAJ01000000, JAAK01000000, JAAL01000000 and JAAM01000000.

## Results

### PFGE patterns and antimicrobial susceptibility of VREfm and VSEfm

**Figure 1:**
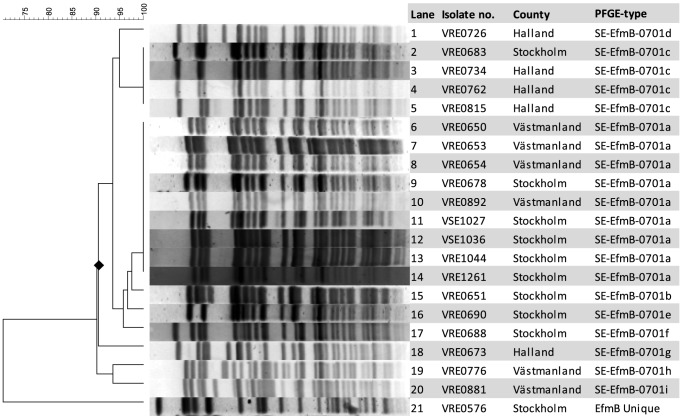
Dendrogram of *Sma*I PFGE of the 21 isolates in collection A. The dendogram shows that 20 of the isolates are clonal (lane 1–20) and one is divergent (lane 21). The symbol ♦ in the dendrogram indicates a similarity of 90.6%. The PFGE-type nomenclature is based on the following: SE stands for Sweden, EfmB stands for *E. faecium* with *vanB*, the number 07 represents year 2007 (the year the index was identified) and the last number is a serial number. The letter at the end describes which PFGE-subtype the isolate belongs to.

In an attempt to identify a putative ancestor of the VREfm outbreak strain, we examined consecutive blood isolates (n = 191, Collection B) of *E. faecium* from Karolinska University Hospital, Huddinge, during a four-year period (2006–2009) covering the time of appearance of the presumably first clinical isolate of the outbreak VREfm strain. A dominant PFGE pattern (n = 37; 26 VSEfm and 11 VREfm) was indistinguishable or closely related to that of the VREfm outbreak strain (named SE-EfmB-0701). Representative isolates with this PFGE-pattern are shown in Figure 1. Among the blood isolates analysed in retrospect, this PFGE pattern was observed for the first time in a vancomycin susceptible isolate (VSEfm) from February 2007 and soon after in two more VSEfm. Three additional VREfm with identical PFGE pattern were detected during the autumn of 2007. The first known clinical isolate (VRE0651) was found in an abdominal infection in August 2007. In 2008 another eight VREfm and 14 VSEfm isolates with the same PFGE pattern were detected. During January 1^st^ until August 31^st^ 2009 no more VREfm but still 10 VSEfm of the SE-EfmB-0701 PFGE type were detected.

Antimicrobial susceptibility testing of the 37 blood culture isolates revealed high-level gentamicin resistance (n = 14; 38%) as well as ampicillin (100%) and ciprofloxacin (100%) resistance and teicoplanin susceptibility (100%). The *vanB* gene was detected in all VREfm isolates.

In collection A (n = 21), representing PFGE patterns as diverse as possible at the time of selection (autumn 2008), a total of 9 subgroups (a to i) of the pattern SE-EfmB-0701 were found (Table 1). All PFGE patterns displayed a similarity >90%, thereby fulfilling the suggested definition of relatedness (>81%) according to Morrison *et al.* [38]. The isolates did however not group together consistently in relation to their geographical origin (Figure 1).

**Table 1:** Demographic data and relevant characteristics for collection A strains.

Isolate ID	County	Material	Year-month of isolation	MLST type	SE-EfmB PFGE type^a^	*vanB2*-Tn*5382*	vanco-mycin MIC	teico-planin MIC	*rep*_17/pRUM_	rep_2/pRE25_	*rep*_pLG1_	Unknown replicons	Mutations involved in CIP resistance, GyrA and ParC		Virulence genes^b^
VSE1036	Stockholm	Blood	2007-02	192	0701a	Neg	1	1	40-kb	50-kb	160-kb	100-kb	S83I	S80R	*hyl*
VSE1027	Stockholm	Blood	2007-06	192	0701a	Neg	2	1	40-kb	50-kb	160-kb	100-kb	NT	NT	*hyl, pilA*
VRE0673	Halland	Faeces	2008-04	78	0701g	Pos	8	0,5	70-kb	50-kb, 70-kb	160-kb	10-kb, 100-kb	NT	NT	*hyl, orf903*
VRE0726	Halland	Wound	2008-07	192	0701d	Pos	≥256	0,5	70-kb	50-kb, 70-kb	160-kb	100-kb	NT	NT	*hyl*
VRE0734	Halland	Faeces	2008-08	192	0701c	Pos	64	1	70-kb	25-kb, 70-kb	160-kb	100-kb	NT	NT	*hyl, pilA*
VRE0762	Halland	Faeces	2008-09	192	0701c	Pos	≥256	1					NT	NT	*hyl, pilA*
VRE0815	Halland	Faeces	2008-11	192	0701c	Pos	16	0,5	70-kb	45-kb, 70-kb	160-kb	100-kb	NT	NT	*hyl*
VRE0651	Stockholm	Abdominal drainage fluid	2007-08	192	0701b	Pos	32	0,5	70-kb	50-kb, 70-kb	160-kb	100-kb	NT	NT	*hyl*
VRE0678	Stockholm	Faeces	2007-11	192	0701a	Pos	16	0,5	70-kb	50-kb, 70-kb	160-kb	100-kb	NT	NT	*hyl*
VRE0683	Stockholm	Faeces	2007-11	192	0701c	Pos	16	1					NT	NT	*hyl*
VRE0688	Stockholm	Faeces	2008-02	192	0701f	Pos	32	1	70-kb	50-kb, 70-kb		100-kb	NT	NT	*pilA*
VRE0690	Stockholm	Faeces	2008-02	192	0701e	Pos	16	0,5	70-kb	50-kb		100-kb	NT	NT	
VRE1044	Stockholm	Blood	2007-12	192	0701a	Pos	32	1	70-kb	50-kb, 70-kb	160-kb	100-kb	S83I	S80R	*hyl*
VRE1261	Stockholm	Blood	2008-10	192	0701a	Pos	16	1	70-kb	50-kb, 70-kb	160-kb	100-kb	S83I	S80R	*hyl*
VRE0576	Stockholm	Blood	2006-03	17	Unique	Pos	64	0,5					S83R	S80I	*hyl*
VRE0650	Västmanland	Urine	2008-01	192	0701a	Pos	32	0,5	70-kb	50-kb, 70-kb	160-kb	100-kb	NT	NT	*hyl*
VRE0653	Västmanland	Urine	2008-02	192	0701a	Pos	16	0,125	70-kb	50-kb, 70-kb		100-kb	NT	NT	
VRE0654	Västmanland	Wound	2008-02	192	0701a	Pos	16	1					NT	NT	*hyl, pilA*
VRE0776	Västmanland	Faeces	2008-10	192	0701h	Pos	≥256	0,25	70-kb	40-kb		70-kb, 100-kb	NT	NT	*hyl, pilA, ecbA*
VRE0881	Västmanland	Faeces	2008-12	17	0701i	Pos	32	0,5	70-kb	25-kb, 70-kb	160-kb	100-kb	NT	NT	*hyl, pilA*
VRE0892	Västmanland	Faeces	2008-12	192	0701a	Pos	≥256	0,25	70-kb	25-kb, 70-kb	160-kb	100-kb	NT	NT	*hyl*

R, resistant; HLR, high level resistant; NT, not tested; ND, not detected, S, serine; I, isoleucine; R, arginine.

All isolates were susceptible to teicoplanin, resistant to ampicillin and high level resistant to ciprofloxacin in addition to the resistance profile shown in this table.

a A 97% threshold similarity value of Dice dendrogram was used to designate PFGE subtype (small letter).

b All isolates were positive for *esp*, *srgA*, *efaAfm*, *acm*, *scm*, *pilB*, *orf2010* and *orf2514* by PCR in addition to the virulence gene results shown in this table. All isolates were also positive for *pilA* and *ecbA* with *pilA 2* and *ecbA 2* primers.

The nineteen VREfm isolates in collection A had vancomycin MICs ranging from 8 to ≥256 mg/L and were susceptible to teicoplanin, consistent with the *vanB2* genotype. All the isolates were resistant to ampicillin and ciprofloxacin, and seven displayed high-level resistance to gentamicin. The two VSE-isolates (VSE1036 and VSE1027) in collection A were susceptible to both vancomycin and teicoplanin, but expressed resistance to ampicillin and ciprofloxacin (Table 1).

### MLST and WGS analyses of VREfm and VSEfm

Collection A (n = 21) were studied in greater detail by MLST and PCR for virulence genes (Table 1). Three isolates of the outbreak strain and one unrelated VREfm isolate were examined by WGS; VSE1036 (ST192), VRE1044 (ST192), VRE1261 (ST192) and VRE0576 (ST17). In collection A, all isolates including the two VSE belonged to ST192 except one single locus (VRE0673, ST78) and two double locus (VRE0881 and VRE0576, ST17) variants of ST192. All isolates except VRE0881 and VRE0576 belonged to the ST78 lineage. All isolates except VRE0576, a pre-outbreak *vanB*-positive ST17 isolate from 2006 had a related PFGE pattern as shown by *Sma*I PFGE (Figure 1). The MLST- and PFGE results concurred moderately in showing relation, since the isolates with the most divergent PFGE patterns (VRE0673, VRE0776 and VRE0881) in two of three cases had deviating MLST profiles.

The WGSs from the outbreak isolates VRE1044, VRE1261, and VSE1036 as well as the unrelated *vanB*-positive pre-outbreak isolate VRE0576 were aligned against the chromosome of an *E. faecium* isolate from Australia which contains a *vanB2* transposon (Aus0004) (Figure S1, *vanB2* transposons are indicated by red triangles). The WGS of the three ST192 outbreak isolates were naturally more homologous and shared several regions (highlighted by black triangles) that were not present in the genome sequences of either VRE0576 or Aus0004 (both ST17) although it should be noted that the plasmid sequences of Aus0004 were not included in this comparison. The VRE0576 and Aus0004 genomes showed many unique regions (white areas). Furthermore, VRE1044 and VRE1261 showed some unique regions (green triangles) that were not present in VSE1036. Further analyses of these regions suggested they belong to mobile genetic elements.VRE1044 and VRE1261 showed only minor differences.

### Antimicrobial resistance determinants and virulence genes of VREfm and VSEfm

All VREfm isolates in collection A harboured the *vanB2* gene as an integral part of Tn*1549*/Tn*5382* demonstrated by the *vanX_B_*-ORFC-PCR (Table 1). This link was also confirmed by WGSs in three VREfm isolates (Table S2). WGS data of VRE576 revealed the same genetic organisation of the *vanB2* transposon as well as 99% nucleotide (nt) identity to Tn*1549*. Interestingly, WGS data from VRE1044 and VRE1261 showed the same *vanB2* transposon organisation and 99% nucleotide identity to Tn*1549*, but also an additional 2588 bp inserted between nt 5014 and 5015 of Tn*1549*. We performed ICE*Slu*van Q8 PCR (Table S1) covering the putative insertion region in the remaining VRE isolates to disclose a potential unique insertion signature of the *vanB* transposon in SE-EfmB-0701 isolates. Presence of an approximately 2.6 kb insertion was confirmed in the *vanB* transposon of all the VREfm SE-EfmB-0701 isolates in collection A. The 2588 bp insert sequence is 89% identical to the region in *Clostridium saccharolyticum-like* K10 (GenBank Acc. No. FP929037) encoding a retron-type reverse transcriptase. In line with this, the 2588 bp sequence encodes a putative protein of 610 amino acids (aa) with 99% identity to a putative reverse transcriptase/maturase from *Faecalibacterium prausnitzii* A2-165 (GenBank Acc. No. EEU96266) and a putative group II intron-encoded protein LtrA (reverse transcriptase and RNA maturase) from *Flavonifractor plautii* ATCC 29863 (GenBank Acc. No. EHM54980). The putative protein further shows 43% identity to the group II intron 599 aa multifunctional protein LtrA in *Lactococcus lactis* (GenBank Acc. No. U50902). LtrA is known to have reverse transcriptase, RNA maturase and site-specific DNA endonuclease activity mediating intron splicing and mobility [39].

The *gyrA* and *parC* genes extracted from the WGSs revealed SNPs associated with ciprofloxacin resistance. Two mutation events in each gene were found (Table 1), and both aa combinations (GyrA Arg83, ParC Ile80 or GyrA Ile83, ParC Arg80) have been described previously in *E. faecium* isolates with ciprofloxacin MICs ≥16 mg/L [25], [40], [41]. Moreover, the tetracycline resistance determinant *tetM* and the macrolide resistance determinant *ermB* were also found in three and four WGS isolates, respectively (Table S2).

All isolates of the PFGE type SE-EfmB-0701 harboured *esp*, *sgrA*, *acm*, *scm*, *pilB*, *efaAfm*, *orf*2010 and *orf* 2514. Moreover, 17 of 20 isolates contained *hyl*. PCR data showed that the genes *pilA*, *ecbA* and *orf*903 occurred in six, one and one of 20 isolates, respectively. The *ecbA* and *orf*903 genes were found in isolates with unique PFGE subtypes in this collection (VRE0776 and VRE0673) (Table 1). WGS data revealed that *pilA* (VRE1044 and VRE1261) and *ecbA* (VSE1036, VRE1044 and VRE1261) were present with a nucleotide match of 1672/1976 (85%) for *pilA* and 2766/3173 (87%) for *ecbA* compared to reference sequences in isolates E1162 and TX16, respectively. In addition, VSE1036, VRE1044, and VRE1261 carried a truncated version of *pilA* (219 bp) 100% identical to the reference sequences. Blastn search of the *pilA* and *ecbA* sequences within the NCBI shotgun sequence database revealed that these two genes had a notable SNP variation between different strains (0–20%). The original *pilA* and *ecbA* primers used yielded no good matches in the WGS sequences which explain why we got few positive PCR products with these primers. All our collection A isolates were positive for these genes using new primers (*pilA 2* and *ecbA 2* Table S1) targeting conserved regions in the WGS and reference *ecbA* and *pilA* genes.

### Plasmid localisation of Tn*1549*-type transposon

**Figure 2:**
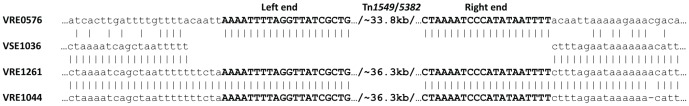
Sequence comparison of the insertion regions of Tn*1549/5382*. The figure shows the transposon insertion regions of VRE0576 versus VRE1044 and VRE1261 and the corresponding region in VSE1036 (contig00062). Tn*1549*/Tn*5382* left and right end imperfect inverted repeats are shown in bold capital letters. Vertical lines indicate identical nucleotides.

By BLAST alignment of the contigs from VRE1044 and VRE1261 containing the *vanB2* transposon against the WGS data of the VSE1036 isolate, the exact AT-rich location of the *vanB2* transposon insertion site could be identified in contig00062 of VSE1036 (Figure 2 and blue triangle in Figure S1). The transposon insertion site was identical for VRE1044 and VRE1261 and corresponded with 100% identity to sequence in contig00062 of VSE1036 (Figure 2).

To get a better picture of the plasmid content we performed Southern blot hybridisation of S1 nuclease treated total DNAs separated by PFGE using specific replication (*rep*) gene probes (Table 1). WGS data gave additional information on plasmid sequences as well as resistance genes and Tn*1549*/Tn*5382* (Table S2). Replication genes of *rep*-classes *rep*_2/pRE25_, *rep*_11/pB82_, *rep*_17/pRUM_ and *rep*_pLG1_ were present in all four WGS, with some nucleotide differences in the pre-outbreak isolate (VRE0576) compared to the other three isolates. VRE0576 also differed from the other isolates by lacking a *rep*_14/pRI1_-class gene present with identical nucleotide identity scores in the other WGSs, and by containing a putative *rep*_unique/pCIZ2_-class gene absent in the other WGSs. Notably, VRE1044 and VRE1261 contained two *rep*_2/pRE25_-sequences with SNP differences on different contigs, consistent with the observed two *rep*_2/pRE25_ plasmids in the hybridisation analyses (Table 1 and S2). The VSE1036 isolate had a similar hybridisation pattern as VRE1044 and VRE1261, but did only have one 50-kb plasmid and one contig with *rep*_2/pRE25_.

**Figure 3:**
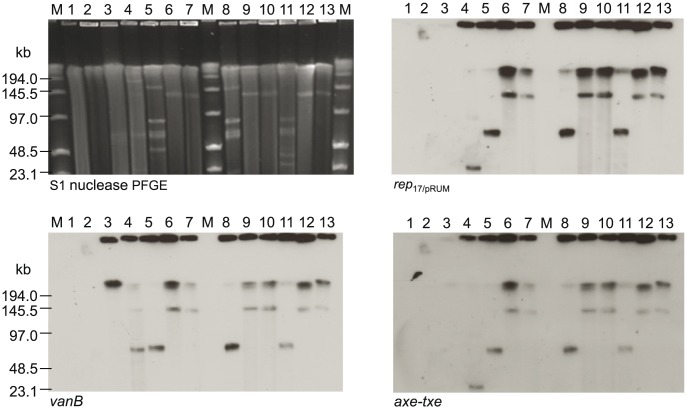
S1-nuclease PFGE and corresponding Southern hybridisations with *rep*_17/pRUM_, *vanB* and *axe-txe* probes. These results illustrated transfer from donors VRE0726, VRE0734 and VRE0881 (lanes 5, 8 and 11) of a similar sized plasmid (approximately 140 kb) to 64/3 (lane 1) (1^st^ generation transconjugants shown in lanes 6, 9 and 12) which was subsequently retransferred to BM4105Str (lane 2) (2^nd^ generation transconjugants shown in lanes 7, 10 and 13) when using the 1^st^ generation transconjugants as donors. Lane 3 *vanB* positive control V583, lane 4 *rep*_17/pRUM_, *axe-txe* and *vanB* positive control *E. faecium* U37, lanes M low-range PFGE marker.

**Figure 4:**
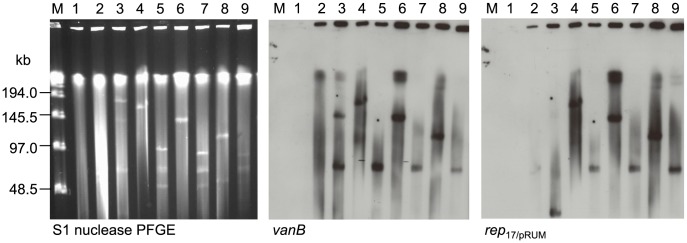
S1-nuclease PFGE and corresponding Southern hybridisations with *vanB* and *rep*_17/pRUM_ probes. Donors VRE0690, VRE0653 and VRE0776 (lanes 5, 7 and 9) and their respective transconjugants (lanes 4, 6 and 8) illustrate transfer of different sized plasmids co-hybridising to *vanB* and *rep*_17/pRUM_ (circa 110–150 kb) into 64/3 (lane 1). Lane 2 *vanB* positive control V583, lane 3 *rep*_17/pRUM_ and *vanB* positive control *E. faecium* U37, lane M low-range PFGE marker.

Hybridisation results showed that all VREfm isolates harboured the *vanB* resistance gene on an approximately 70-kb *rep*_17/pRUM_ replicon (shown for representative isolates VRE0726, VRE0734 and VRE0881 in Figure 3 lanes 5, 8 and 11, and VRE0690, VRE0653, VRE0776 in Figure 4 lanes 5, 7 and 9). Further hybridisation of selected isolates revealed a dominant common plasmid content pattern for VRE0726, VRE0651, VRE0678, VRE1044, VRE1261, and VRE0650, and some minor differences in the other 11 characterised isolates (Table 1).

Interestingly, the two VSE isolates clonally related to the VREfm outbreak strain (VSE1027 and VSE1036) differed from the common pattern by having a *rep*_17/pRUM_ replicon 30 kb smaller than that of the VREfm isolates (Table 1), corresponding to the size of the *vanB2*-Tn*1549*/Tn*5382* transposon. Notably, the pRUM *repA* was found with 100% homology to the reference sequence in VRE0576 (GenBank Accession number JAAK00000000), and present with 97% homology in VSE1036 (JAAJ00000000), VRE1044 (JAAL00000000) and VRE1261 (JAAM00000000). A subsequent comparison of the *rep*_17/pRUM_ replicon sequence in the three latter strains showed 100% homology. Thus, these plasmids share identical signature sequences for pRUM *repA* as well as for the Tn*1549*/Tn*5382* transposon insertion site.

Tn*1549*/Tn*5382*-related genes, *axe-txe* and the *vanB2* gene were found within the same contig in the VRE1044 and VRE1261 isolates, thus supporting the S1 nuclease PFGE hybridisation and PCR data showing linkage of *vanB2*-Tn*1549*/Tn*5382* and *axe-txe* genes on the same replicon (representative isolates in Figure 3 lanes 5, 8 and 11). WGS analyses of VSE1036 showed that the sequence corresponding to the transposon insertion site of VRE1044 and VRE1261 (Figure 2) mapped to the same contig00062 as *axe-txe* (Table S2) typically found on *rep*_17/pRUM_ replicons. Interestingly, the reference pRUM plasmid contains a putative relaxase gene and a mobilisation gene both consistently absent from all four WGSs.

### Transferability studies of the VanB determinant

All but 2 (VRE0651 and VRE0673) out of 10 tested VREfm isolates produced transconjugants with a low frequency of 10^−8^ to 10^−11^ per donor, close to the detection limit, using *E. faecium* 64/3 as a recipient (Table 2). Transconjugants were confirmed by PFGE using *Sma*I-digestion (Figure S2 and S3). *E. faecium* recipient BM4105-RF did not support conjugation within the detection limit.

**Table 2:** Transfer frequencies between donors and recipients after filter mating.

Primary matings		Transfer frequency	
Donor (PFGE subtype) (ST)	Recipient	Transconjugants/donor	Transconjugants/recipient
VRE0653 (a) (192)	64/3	6×10^−9^	4×10^−10^
VRE0651 (b) (192)	64/3	<6×10^−9^ a	<2×10^−9^
VRE0683 (c) (192)	64/3	8×10^−9^	5×10^−11^
VRE0734 (c) (192)	64/3	2×10^−11^	2×10^−12^
VRE0726 (d) (192)	64/3	1×10^−9^	3×10^−11^
VRE0690 (e) (192)	64/3	2×10^−9^	1×10^−10^
VRE0688 (f) (192)	64/3	7×10^−9^	6×10^−9^
VRE0673 (g) (78)	64/3	<2×10^−8^ a	<1×10^−9^
VRE0776 (h) (192)	64/3	1×10^−10^	5×10^−8^
VRE0881 (i) (17)	64/3	2×10^−8^	7×10^−11^

Secondary matings			
VRE0726×64/3	BM4105-Str	7×10^−5^	2×10^−3^
VRE0734×64/3	BM4105-Str	1×10^−3^	2×10^−3^
VRE0881×64/3	BM4105-Str	3×10^−4^	1×10^−3^

a Isolates VRE0651 and VRE0673 did not support conjugation within given detection limits.

S1 nuclease PFGE and subsequent hybridisation of the 64/3 transconjugants revealed that all the transconjugants contained a *vanB*-*rep*_17/pRUM_ plasmid of variable size (110–170 kb), larger than the original 70-kb donor plasmids (Figures 3, 4, S5 and S6). With the exception of VRE0690×64/3 (170 kb) (Figure 4 lane 4 and Figure S5 lane 7) and VRE0776×64/3 (110 kb) (Figure 4 lane 8 and Figure S5 lane 9), the transconjugants had a *vanB*-*rep*_17/pRUM_-*rep*_2/pRE25_ containing plasmid of about 140 kb (Figure 3 lanes 6, 9 and 12, Figure 4 lane 6, Figure S5 lanes 3, 5 and 11 and Figure S6 lanes 5, 8 and 11) which corresponds to the joint size of the 70-kb *rep*_2/pRE25_ and the 70-kb *vanB*-*rep*_17/pRUM_ of their donors. Conversely, the two donors VRE0690 and VRE0776 that gave transconjugants with different sized plasmids both lacked a copy of the 70-kb *rep*_2/pRE25_ plasmid (Table 1). VRE0776 contain a 40-kb *rep*_2/pRE25_ plasmid which joint with the 70-kb *vanB*-*rep*_17/pRUM_ could result in the 110-kb *vanB*-*rep*_17/pRUM_-*rep*_2/pRE25_ seen in Figure S5 lane 9. In VRE0690 the 170 kb plasmid of the transconjugant did not hybridise to *rep*_2/pRE25_ suggesting that the 170-kb plasmid result from joining of the 70-kb *vanB*-*rep*_17/pRUM_ with the 100-kb unknown replicon (Figure S5 lane 7).

The transconjugants were tested for susceptibility to streptomycin in order to identify eligible donors for retransfer experiments. The first generation transconjugants originating from donors VRE0726 (ST192), VRE0734 (ST192) and VRE0881 (ST17) were susceptible. The streptomycin resistance in the other transconjugants probably originate from co-transfer of streptomycin resistance with the plasmids of their clinical isolate donors (VRE0653, VRE0683, VRE0690, VRE0688 and VRE0776) that all showed high-level resistance to streptomycin.

Retransfer and following S1 nuclease PFGE and Southern hybridisations demonstrated that the *vanB*-*rep*_17/pRUM_-*rep*_2/pRE25_ 140-kb hybrid plasmids (Figure 3 and Figure S6) were stable in size and readily transferable with transfer rates of 10^−3^–10^−5^ transconjugants per donor (Table 2). Transconjugants were confirmed by PFGE using *Sma*I-digestion (Figure S4).

## Discussion

A total of 872 VREfm cases were notified during the outbreak in 2007 to 2011, predominantly as faecal colonisation in elderly hospitalised patients with underlying diseases. Less than 10% of the VREfm isolates were recorded from blood, urine or wound samples [15].

Molecular characterisation of a representative subset of related outbreak isolates showed a strong predominance of ST192, which is a single-locus variant of ST78 and considered to be a high-risk genotype [42]. The MLST- and PFGE results were moderately congruent, as the isolates with less similar PFGE patterns and virulence profiles (VRE0673, VRE0776 and VRE0881) in two of three cases had a divergent MLST profile. Previous BAPS data have concluded that the ST78 and ST17 lineages are located in BAPS group 2-1 and 3-3, respectively, and not closely related. This contrasts our PFGE results which grouped VRE0881 (ST17 lineage) in the SE-EfmB-0701 PFGE type (ST78 lineage). This observation as well as the carriage of a unique virulence gene by VRE0673 (*orf903*) and VRE0776 (only *ecbA* gene detected by the original PCR primers), could be explained by the lean inclusion criteria of relatedness by PFGE, thereby accepting not genetically related pulsotypes as related. The long collection time (around a year) and evidence of increased DNA banding pattern polymorphism by *E. faecium* compared to other bacteria [38] was used as basis for our choice of inclusion criteria. However, the unique *vanB* transposon signature found in all the SE-EfmB-0701 isolates links VRE0881, VRE0776 and VRE0673 to the outbreak.

The examined isolates expressed multidrug-resistance and harboured several specific genes associated with increased virulence. The vancomycin susceptible isolates (VSE1027 and VSE1036) from the start of the outbreak period were clonally related and exerted the same co-resistance- and virulence profile as the VREfm isolates (Figure 1 and Table 1). Moreover, plasmid profiling and WGS data (Table 1 and S2, Figure S1) also indicated close relatedness between SE-EfmB-0701 PFGE type isolates with minor differences in plasmid profile as well as clear differences in gene content compared to the pre-outbreak isolate.

Our results support the notion that internalisation of the *vanB* transposon into the *rep*_17/pRUM_ plasmid coincide with the successful spread of this high-risk strain. The *rep*_17/pRUM_ replicon has previously been shown to harbour a segregation stability module encoded by a toxin-antitoxin cassette (*axe-txe*) which have been shown to support maintenance of linked antimicrobial resistance genes [43]. Notably, *rep*_17/pRUM_ replicons with the *axe*-*txe* cassette have been shown to be present in a majority of CC17-like strains [22]. WGS analyses revealed that the SE-EfmB-0701 pRUM replicons contained an *axe-txe* module with a 100% identity to the original pRUM *axe-txe* sequence.

A *rep*_17/pRUM_-*vanB2*-Tn*1549*/Tn*5382-axe-txe-*plasmid of approximately 120–130 kb has previously been described in a polyclonal cluster of *E. faecium* from 2002–2004 in the Swedish county Örebro. This cluster originated from BAPS-group 3-3 (ST17, ST18 and single locus variants of these) [18]. The Örebro isolates displayed a different PFGE pattern from SE-EfmB-0701 [15] and had a *vanB2* transposon without the unique signature found in SE-EfmB-0701.

The repeatedly observed fusion between the *vanB2*-containg *rep*_17/pRUM_ plasmids and *rep*_2/pRE25_ replicons during conjugation experiments is an interesting feature. The WGS data did not support the presence of the putative relaxase and mobilisation protein associated with the reference pRUM plasmid. This could explain the need for the *vanB2*-containing *rep*_17/pRUM_ plasmids to fuse with a conjugation system from other intracellular sources in order to be mobilised. Several studies have described mosaicism and/or recombination events between enterococcal plasmids [22], [44] which may support enhanced host range or other functional benefits associated with several replicons in one plasmid.

There is a noteworthy reservoir of *vanB* in intestinal anaerobes, and introduction of *vanB2-*Tn*1549/*Tn*5382* in enterococci from other co-habitants (mainly Gram-positive anaerobes) in the intestinal environment has been experimentally observed [45]. Howden *et al.* [46] tested the ecological impact by phylogenetic analysis of the transposons and their insertion sites, and showed that a diversification was likely due to a higher grade of *de novo* VRE generation compared to cross-transmission between enterococcal strains than previously believed. They also observed an increasing incidence of nosocomial VRE infections despite engagement of control interventions to limit transmission between patients. Based on the extensive PFGE and selective MLST analyses in this study, it is highly probable that the closely related VSEfm ST192 strain was a successful hospital coloniser in Sweden already in 2007. Acquisition of the *vanB2* transposon by the VSEfm ST192 outbreak strain is the most likely hypothesis on how vancomycin resistance appeared in this strain. The WGS data strongly support that a *vanB2* transposon with unique signature was inserted within a *rep*_17/pRUM_-plasmid with an unique pRUM *repA* sequence signature in a strain already present, causing a parallel evolution between VSEfm clones without, and VREfm clones with the *rep*_17/pRUM_-*vanB2*-Tn*1549*/Tn*5382-axe-txe* arrangement.

Suggested clearance time for VRE faecal colonisation is estimated to be 4 years [47], but others have suggested that environmentally adapted VRE are capable of inhabiting the intestines in small numbers for even longer [48], [49].

The selective enriched broth used in some laboratories in Sweden before January 2009 with a vancomycin concentration of 32 mg/L was not suitable for *vanB-*type resistance screening. To address this problem, microbiological laboratories were then advised to reduce the concentration to 4 mg/L [15]. However, *vanB*-type VRE may have even lower MICs [17], [50]. Importantly, the EUCAST disk diffusion test used by most laboratories in Sweden as the phenotypic vancomycin-susceptibility test method rely on observing the zone edge quality for identification of low level *vanB*-type resistance. This introduces observer experience as a variable [51].

In conclusion, the molecular analyses revealed that the *E. faecium* outbreak strain belonged to the high-risk genetic lineage of ST192. The strain was resistant to several commonly used antibiotics and harboured several virulence genes. A successful *rep*_17/pRUM_-plasmid containing a *vanB* transposon with a unique genetic signature originating from other intestinal bacterial species was present in all the VREfm isolates related to the outbreak strain. The *rep*_17/pRUM_ plasmid harboured a toxin-antitoxin module supporting plasmid maintenance. In addition the *rep*_17/pRUM_ replicon can easily join with conjugative genetic elements supporting spread to other high-risk *E. faecium* clones. The current phenotypic screening methods might hamper efforts in limiting VREfm spread, as low-MIC *vanB*-type VRE might go undetected [51].

## Supporting Information

Figure S1 Alignment of genome sequences from VRE1044 (row 2), VRE1261 (row 3), VSE1036 (row 4) and VRE0576 (row 5) against the reference genome Aus0004 NC_017022 (row 1). The similarity plot indicates average similarity for each region. Coloured blocks indicate regions of sequence homology in the genomes and white areas indicate regions with low sequence homology. Red triangles indicate contigs containing Tn*1549*/Tn*5382* (VRE1044 contigs 00036 and 00041, VRE1261 contigs 00049 and 00044/VRE0576 contig 00004). The blue triangle indicates the transposon Tn*1549*/Tn*5382* insertion region in contig 00062 of VSE1036. This contig also contains the *axe-txe* genes typically found on *rep*_17/pRUM_ replicons. Black (VRE1044, VRE1261 and VSE1036) and green (VRE1044 and VRE1261) triangles highlight regions with contigs or partial contigs found in the outbreak isolates but not in VRE576 or Aus0004.
(PDF)

Figure S2 *Sma*I PFGE of first generation transconjugants (TC) (lanes 5, 7, 9, 11) showing divergent band patterns compared with the clinical isolate donors (lanes 6, 8, 10 and 12) and similar pattern with recipient 64/3 (lane 2). Lanes 1 and 13 low-range PFGE marker, lane 3 *vanB* positive control *E. faecalis* V583, lane 4 *rep*_17/pRUM_ positive control *E. faecium* U37, lanes 5 and 6 TC and donor VRE0726, lanes 7 and 8 TC and donor VRE0734, lanes 9 and 10 TC and donor VRE0683, lanes 11 and 12 TC and donor VRE0688.
(PDF)

Figure S3 *Sma*I PFGE of first generation transconjugants (TC) (lanes 5, 7, 9, 11) showing divergent band patterns compared with the clinical isolate donors (lanes 6, 8, 10 and 12) and similar pattern with recipient 64/3 (lane 2). Lanes 1 and 13 low-range PFGE marker, lane 3 *vanB* positive control *E. faecalis* V583, lane 4 *rep*_17/pRUM_ positive control *E. faecium* U37, lanes 5 and 6 TC and donor VRE0690, lanes 7 and 8 TC and donor VRE0653, lanes 9 and 10 TC and donor VRE0776, lanes 11 and 12 TC and donor VRE0881.
(PDF)

Figure S4 *Sma*I PFGE of second generation transconjugants (TCs) (lanes 6–8, 11–13 and 15–17) showing divergent band patterns compared with the first generation transconjugant donors (lanes 5, 10 and 14) and similar pattern with recipient BM4105-Str (lane 4). Lanes 1, 9 and 18 low-range PFGE marker, lane 2 *vanB* positive control *E. faecalis* V583, lane 3 *rep*_17/pRUM_ positive control *E. faecium* U37, lane 5 donor VRE0726×64/3, lanes 6–8 TCs VRE0726×64/3xBM4105-Str, lane 10 donor VRE0734×64/3, lanes 11–13 TCs VRE0734×64/3xBM4105-Str, lane 14 donor VRE0881×64/3, lanes 15–17 TCs VRE0881×64/3xBM4105-Str.
(PDF)

Figure S5 S1-nuclease PFGE and corresponding Southern hybridisations with *rep*_2/pRE25_ and *rep*_17/pRUM_ probes showing co-hybridisation in first generation transconjugants (lanes 3, 5, 9 and 11). Lanes 1 and 12 low-range PFGE marker, lanes 2 and 3 donor and TC VRE0683, lanes 4 and 5 donor and TC VRE0688, lanes 6 and 7 donor and TC VRE0690, lanes 8 and 9 donor and TC VRE0776, lanes 10 and 11 donor and TC VRE0653.
(PDF)

Figure S6 S1-nuclease PFGE and corresponding Southern hybridisations with *rep*_2/pRE25_ and *rep*_17/pRUM_ probes showing co-hybridisation in first (lane 5, 8 and 11) and second generation transconjugants (lane 6, 9 and 12). Lane M low-range PFGE marker, lane 1 *rep*_17/pRUM_ and *rep*_2/pRE25_ positive control *E. faecium* U37, lane 2 recipient 64/3, lane 3 VRE1044, lane 4 VRE0726, lane 5 VRE0726×64/3, lane 6 VRE0726×64/3xBM4105-Str, lane 7 VRE0734, lane 8 VRE0734×64/3, lane 9 VRE0734×64/3xBM4105-Str, lane 10 VRE0881, lane 11 VRE0881×64/3, lane 12 VRE0881×64/3xBM4105-Str, lane 13 recipient BM4105-Str.
(PDF)

Table S1 Primers used in this article.
(PDF)

Table S2 Plasmid replication, resistance, toxin-antitoxin system and conjugative transposon genes found in the WGSs of the pre-outbreak isolate VRE576 and the three outbreak isolates VSE1036, VRE1044 and VRE1261. Gene identity refers to the reference sequence.
(PDF)
