# Supplementary material for: A Multicentre Hospital Outbreak in Sweden Caused by Introduction of a vanB2 Transposon into a Stably Maintained pRUM-Plasmid in an Enterococcus faecium ST192 Clone
Source: PLoS One. 2014 Aug 25;9(8):e103274. doi: 10.1371/journal.pone.0103274 (PMC4143159; doi:10.1371/journal.pone.0103274)
Supplement: Figure S1 — Alignment of genome sequences from VRE1044 (row 2), VRE1261 (row 3), VSE1036 (row 4) and VRE0576 (row 5) against the reference genome Aus0004 NC_017022 (row 1). The similarity plot indicates average similarity for each region. Coloured blocks indicate regions of sequence homology in the genomes and white areas indicate regions with low sequence homology. Red triangles indicate contigs containing Tn1549/Tn5382 (VRE1044 contigs 00036 and 00041, VRE1261 contigs 00049 and 00044/VRE0576 contig 00004). The blue triangle indicates the transposon Tn1549/Tn5382 insertion region in contig 00062 of VSE1036. This contig also contains the axe-txe genes typically found on rep 17/pRUM replicons. Black (VRE1044, VRE1261 and VSE1036) and green (VRE1044 and VRE1261) triangles highlight regions with contigs or partial contigs found in the outbreak isolates but not in VRE576 or Aus0004. (PDF) [file pone.0103274.s001.pdf]

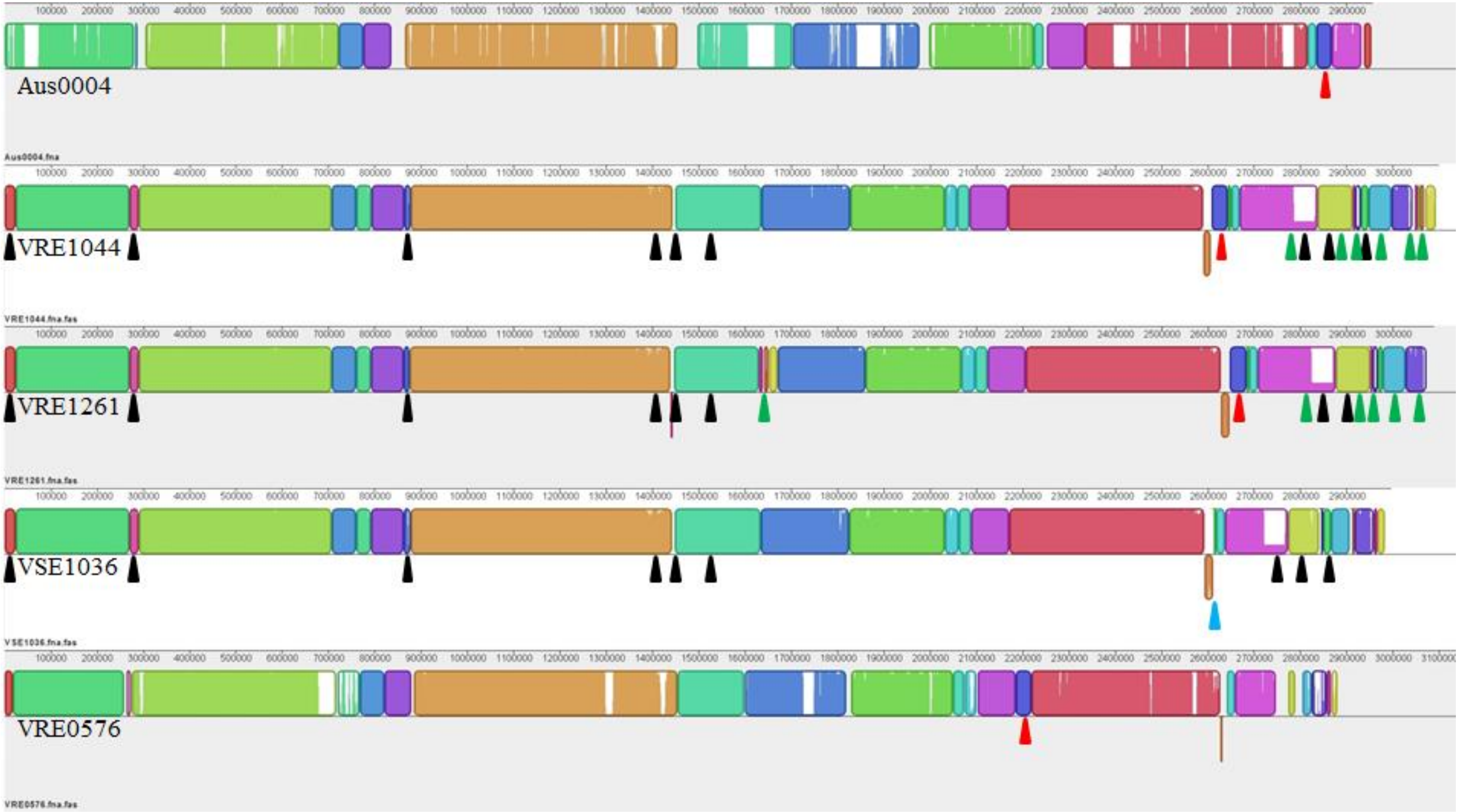

Figure S1. Alignment of genome sequences from VRE1044 (row 2), VRE1261 (row 3), VSE1036 (row 4) and VRE0576 (row 5) against the reference genome Aus0004 NC_017022 (row 1). The similarity plot indicates average similarity for each region. Coloured blocks indicate regions of sequence homology in the genomes and white areas indicate regions with low sequence homology. Red triangles indicate contigs containing Tn*1549*/Tn*5382* (VRE1044 contigs 00036 and 00041, VRE1261 contigs 00049 and 00044/VRE0576 contig 00004). The blue triangle indicates the transposon Tn*1549*/Tn*5382* insertion region in contig 00062 of VSE1036. This contig also contains the *axe-txe* genes typically found on $rep_{17/pRUM}$ replicons. Black (VRE1044, VRE1261 and VSE1036) and green (VRE1044 and VRE1261) triangles highlight regions with contigs or partial contigs found in the outbreak isolates but not in VRE576 or Aus0004.
