# Supplementary material for: A Multicentre Hospital Outbreak in Sweden Caused by Introduction of a vanB2 Transposon into a Stably Maintained pRUM-Plasmid in an Enterococcus faecium ST192 Clone
Source: PLoS One. 2014 Aug 25;9(8):e103274. doi: 10.1371/journal.pone.0103274 (PMC4143159; doi:10.1371/journal.pone.0103274)
Supplement: Figure S2 — SmaI PFGE of first generation transconjugants (TC) (lanes 5, 7, 9, 11) showing divergent band patterns compared with the clinical isolate donors (lanes 6, 8, 10 and 12) and similar pattern with recipient 64/3 (lane 2). Lanes 1 and 13 low-range PFGE marker, lane 3 vanB positive control E. faecalis V583, lane 4 rep 17/pRUM positive control E. faecium U37, lanes 5 and 6 TC and donor VRE0726, lanes 7 and 8 TC and donor VRE0734, lanes 9 and 10 TC and donor VRE0683, lanes 11 and 12 TC and donor VRE0688. (PDF) [file pone.0103274.s002.pdf]

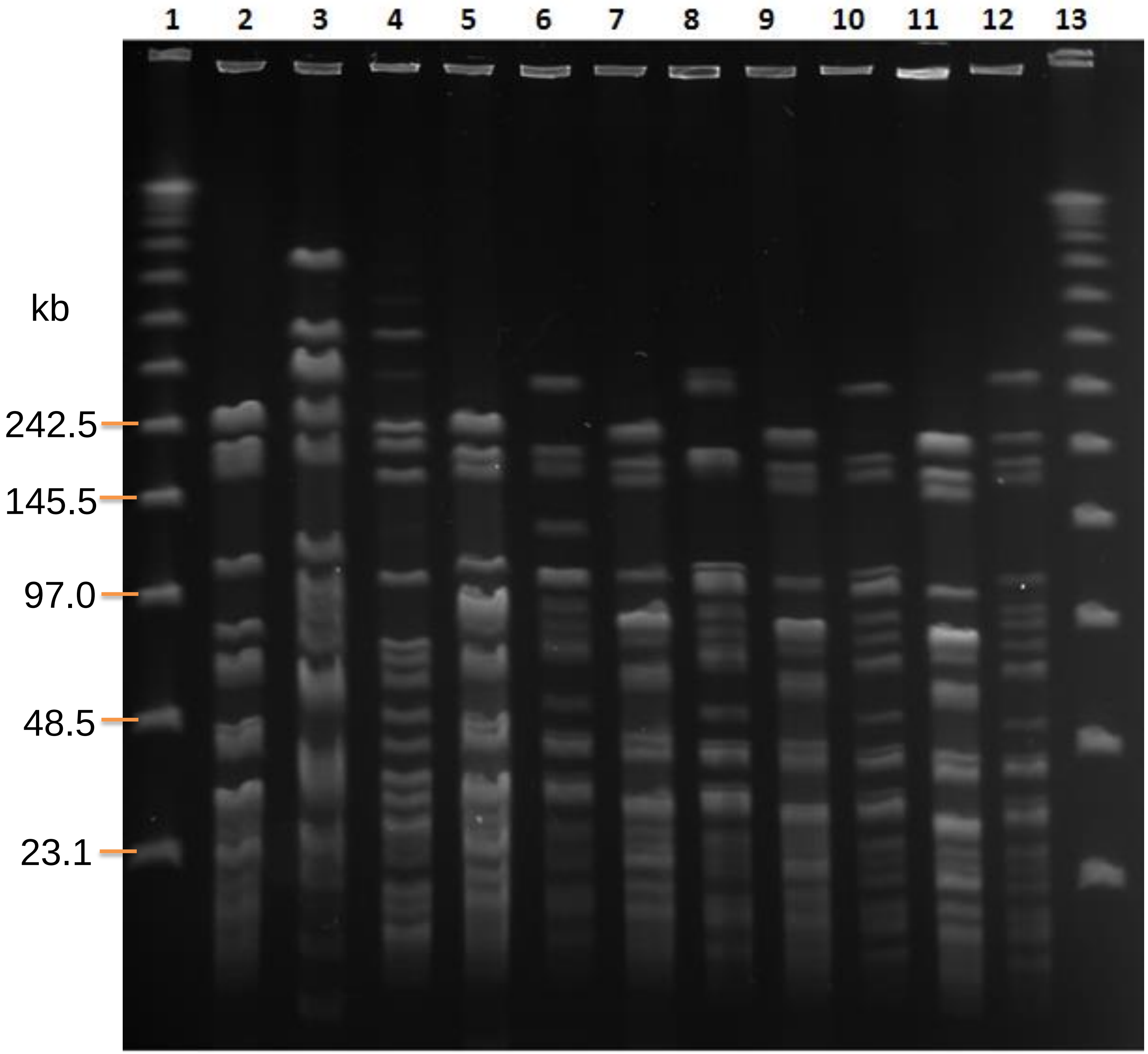

Figure S2. *Sma*I PFGE of first generation transconjugants (TC) (lanes 5, 7, 9, 11) showing divergent band patterns compared with the clinical isolate donors (lanes 6, 8, 10 and 12) and similar pattern with recipient 64/3 (lane 2). Lanes 1 and 13 low-range PFGE marker, lane 3 *vanB* positive control *E. faecalis* V583, lane 4 $rep_{17/pRUM}$ positive control *E. faecium* U37, lanes 5 and 6 TC and donor VRE0726, lanes 7 and 8 TC and donor VRE0734, lanes 9 and 10 TC and donor VRE0683, lanes 11 and 12 TC and donor VRE0688.
