# Supplementary material for: A Multicentre Hospital Outbreak in Sweden Caused by Introduction of a vanB2 Transposon into a Stably Maintained pRUM-Plasmid in an Enterococcus faecium ST192 Clone
Source: PLoS One. 2014 Aug 25;9(8):e103274. doi: 10.1371/journal.pone.0103274 (PMC4143159; doi:10.1371/journal.pone.0103274)
Supplement: Figure S4 — SmaI PFGE of second generation transconjugants (TCs) (lanes 6–8, 11–13 and 15–17) showing divergent band patterns compared with the first generation transconjugant donors (lanes 5, 10 and 14) and similar pattern with recipient BM4105-Str (lane 4). Lanes 1, 9 and 18 low-range PFGE marker, lane 2 vanB positive control E. faecalis V583, lane 3 rep 17/pRUM positive control E. faecium U37, lane 5 donor VRE0726×64/3, lanes 6–8 TCs VRE0726×64/3xBM4105-Str, lane 10 donor VRE0734×64/3, lanes 11–13 TCs VRE0734×64/3xBM4105-Str, lane 14 donor VRE0881×64/3, lanes 15–17 TCs VRE0881×64/3xBM4105-Str. (PDF) [file pone.0103274.s004.pdf]

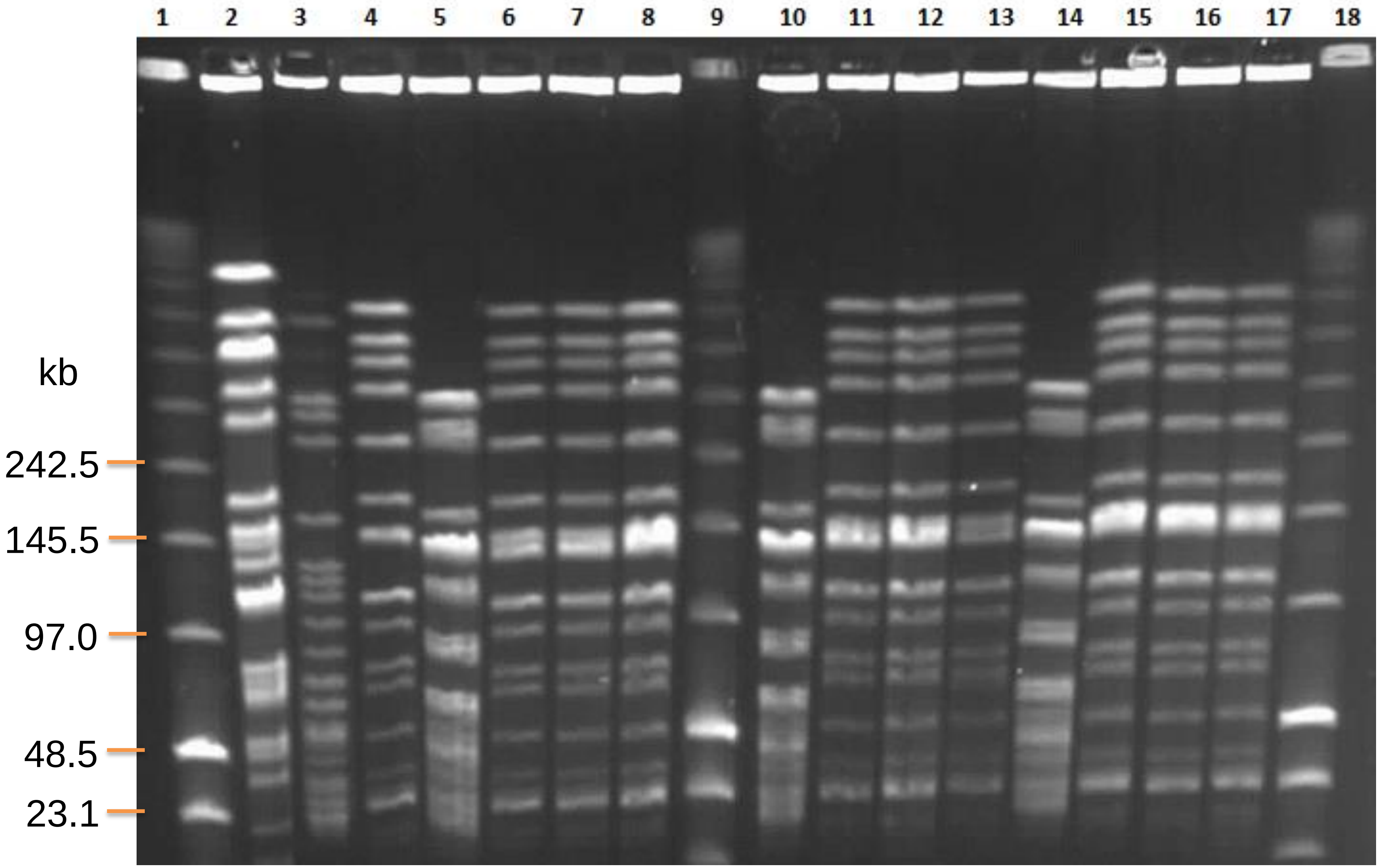

Figure S4. ***Sma*****I PFGE of second generation transconjugants (TCs) (lanes 6-8, 11-13 and 15-17) showing divergent band patterns compared with the first generation transconjugant donors (lanes 5, 10 and 14) and similar pattern with recipient BM4105-Str (lane 4).** Lanes 1, 9 and 18 low-range PFGE marker, lane 2 *vanB* positive control *E. faecalis* V583, lane 3 $rep_{17/pRUM}$ positive control *E. faecium* U37, lane 5 donor VRE0726x64/3, lanes 6-8 TCs VRE0726x64/3xBM4105-Str, lane 10 donor VRE0734x64/3, lanes 11-13 TCs VRE0734x64/3xBM4105-Str, lane 14 donor VRE0881x64/3, lanes 15-17 TCs VRE0881x64/3xBM4105-Str.
