# Supplementary material for: A Multicentre Hospital Outbreak in Sweden Caused by Introduction of a vanB2 Transposon into a Stably Maintained pRUM-Plasmid in an Enterococcus faecium ST192 Clone
Source: PLoS One. 2014 Aug 25;9(8):e103274. doi: 10.1371/journal.pone.0103274 (PMC4143159; doi:10.1371/journal.pone.0103274)
Supplement: Figure S5 — S1-nuclease PFGE and corresponding Southern hybridisations with rep 2/pRE25 and rep 17/pRUM probes showing co-hybridisation in first generation transconjugants (lanes 3, 5, 9 and 11). Lanes 1 and 12 low-range PFGE marker, lanes 2 and 3 donor and TC VRE0683, lanes 4 and 5 donor and TC VRE0688, lanes 6 and 7 donor and TC VRE0690, lanes 8 and 9 donor and TC VRE0776, lanes 10 and 11 donor and TC VRE0653. (PDF) [file pone.0103274.s005.pdf]

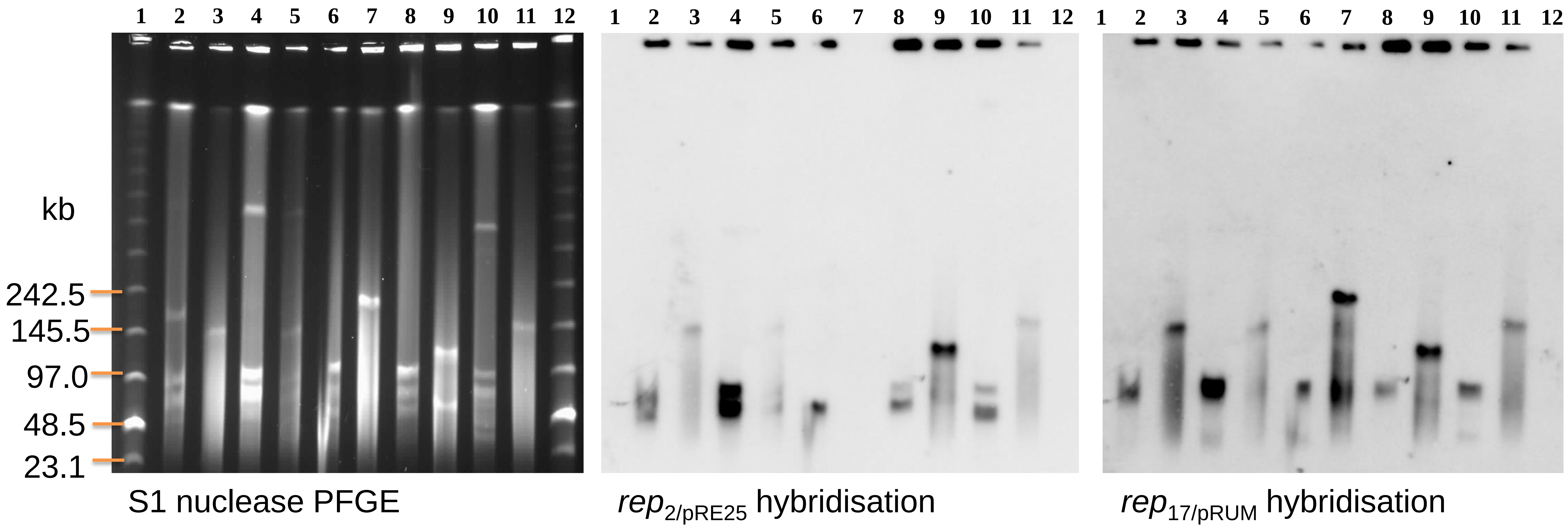

Figure S5. S1-nuclease PFGE and corresponding Southern hybridisations with *rep*$_{2/pRE25}$ and *rep*$_{17/pRUM}$ probes showing co-hybridisation in first generation transconjugants (lanes 3, 5, 9 and 11). Lanes 1 and 12 low-range PFGE marker, lanes 2 and 3 donor and TC VRE0683, lanes 4 and 5 donor and TC VRE0688, lanes 6 and 7 donor and TC VRE0690, lanes 8 and 9 donor and TC VRE0776, lanes 10 and 11 donor and TC VRE0653.
