# Supplementary material for: A Multicentre Hospital Outbreak in Sweden Caused by Introduction of a vanB2 Transposon into a Stably Maintained pRUM-Plasmid in an Enterococcus faecium ST192 Clone
Source: PLoS One. 2014 Aug 25;9(8):e103274. doi: 10.1371/journal.pone.0103274 (PMC4143159; doi:10.1371/journal.pone.0103274)
Supplement: Figure S6 — S1-nuclease PFGE and corresponding Southern hybridisations with rep 2/pRE25 and rep 17/pRUM probes showing co-hybridisation in first (lane 5, 8 and 11) and second generation transconjugants (lane 6, 9 and 12). Lane M low-range PFGE marker, lane 1 rep 17/pRUM and rep 2/pRE25 positive control E. faecium U37, lane 2 recipient 64/3, lane 3 VRE1044, lane 4 VRE0726, lane 5 VRE0726×64/3, lane 6 VRE0726×64/3xBM4105-Str, lane 7 VRE0734, lane 8 VRE0734×64/3, lane 9 VRE0734×64/3xBM4105-Str, lane 10 VRE0881, lane 11 VRE0881×64/3, lane 12 VRE0881×64/3xBM4105-Str, lane 13 recipient BM4105-Str. (PDF) [file pone.0103274.s006.pdf]

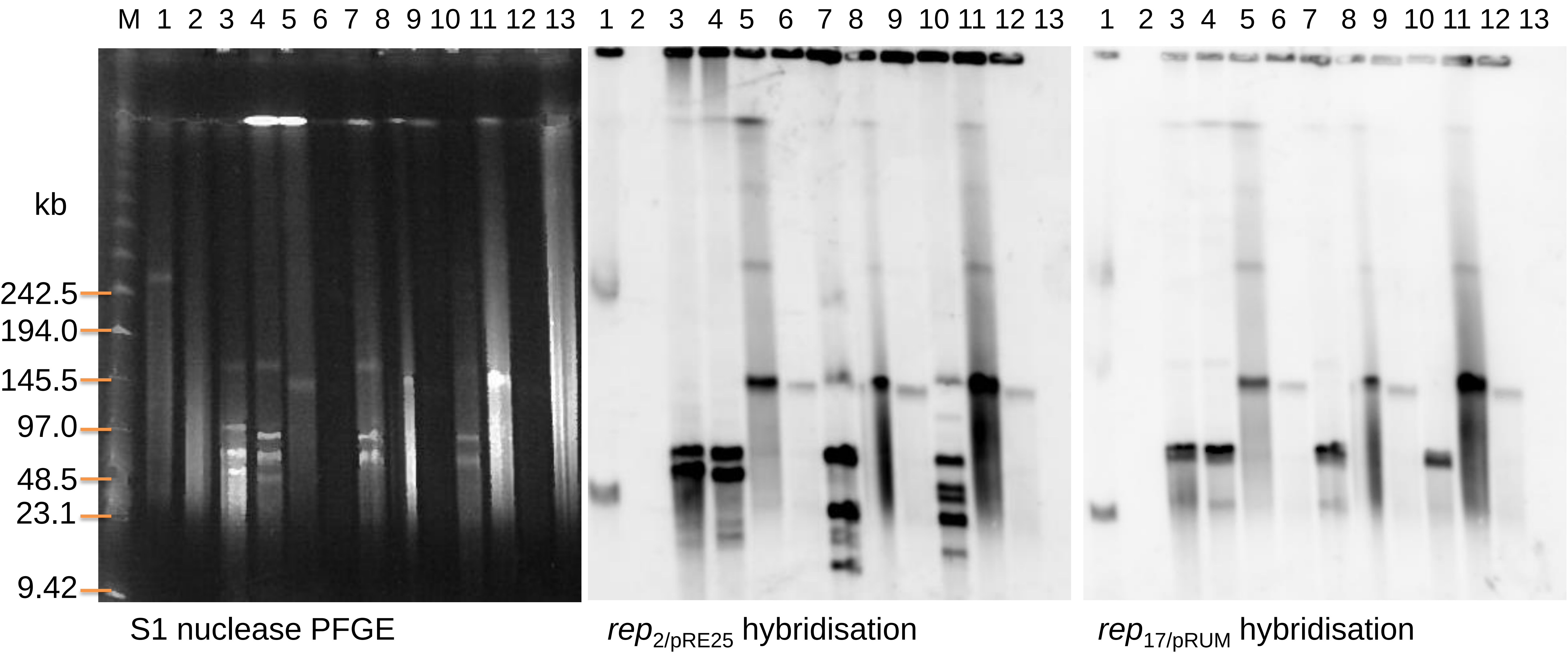

Figure S6. S1-nuclease PFGE and corresponding Southern hybridisations with *rep*$_{2/pRE25}$ and *rep*$_{17/pRUM}$ probes showing co-hybridisation in first (lane 5, 8 and 11) and second generation transconjugants (lane 6, 9 and 12). Lane M low-range PFGE marker, lane 1 *rep*$_{17/pRUM}$ and *rep*$_{2/pRE25}$ positive control *E. faecium* U37, lane 2 recipient 64/3, lane 3 VRE1044, lane 4 VRE0726, lane 5 VRE0726x64/3, lane 6 VRE0726x64/3xBM4105-Str, lane 7 VRE0734, lane 8 VRE0734x64/3, lane 9 VRE0734x64/3xBM4105-Str, lane 10 VRE0881, lane 11 VRE0881x64/3, lane 12 VRE0881x64/3xBM4105-Str, lane 13 recipient BM4105-Str.
