# Supplementary material for: A Multicentre Hospital Outbreak in Sweden Caused by Introduction of a vanB2 Transposon into a Stably Maintained pRUM-Plasmid in an Enterococcus faecium ST192 Clone
Source: PLoS One. 2014 Aug 25;9(8):e103274. doi: 10.1371/journal.pone.0103274 (PMC4143159; doi:10.1371/journal.pone.0103274)
Supplement: Table S1 — Primers used in this article. (PDF) [file pone.0103274.s007.pdf]

Table S1. Primers used in this article

**PCR target:**	**Primer sequence (5'-3')**	**Amplicon size (bp)**	**Annealing temp. (^{o}C)**	**Positive control**	**Reference**
acm	TGACGAGCGGTGATAAAACAGCTA<br>ATAGGCTGTTCATCTGCTCGTCTTA	636	53	TUH7-15 [1]	[2]
efaAfm	GTTCGATAACTTGATGGAAAC<br>CATCTGATAGTAAGAATCTCCTTG	561	53	TUH7-15	[3]
esp	AGATTTCATCTTTGATTCTTGG<br>AATTGATTCTTTAGCATCTGG	510	55	TUH7-15	[4]
hyl	GTTAGAAGAAGTCTGGAAACCG<br>TGCTAAGATATTCCTCTACTCG	Ca 500	53	TUH7-15	[5]
***srgA* (orf2351)**	AATGAACGGGCAAATGAG<br>CTTTTGTTCCTTAGTTGGTATGA	671	50	TUH7-15	[6]
***ecbA* (orf2430)**	GCAGTTTACAATGGTGTGAAGCAA<br>CGGCTAATGAGTATTTGTCGTTCC	963	55	TUH7-15	[6]
ecbA 2	GGTTGGACTGTCTTTGCGAATGGC<br>TGGCCGATTTACAATGAGTTCACTC	951	54	VRE1044	This study
***scm* (orf418)**	CTAACTGGTAACTATGGCTTGT<br>GTCCGTGCTGTCACTTGT	1109	55	TX16 [7]	[6]
***pilA* (orf1904)**	AGGCAGATTATGGTGATGTT<br>GGCTGTTGGTTCTTTATCTG	619	55	TX16	[6]
pilA 2	TGGTTGATCGGCAAATGTAA<br>AGCAGATTATGGGGACGTTG	211	54	VRE1044	This study
***pilB* (orf2569)**	GTGTTTGCAGAGGAGACAGC<br>GACAGAATAATTTACTGGGTCG	1121	55	TX16	[6]
**orf903 (fms11)**	TCAACGGACATACCATACCA<br>CTTACCATCAACGATCTGCC	409	55	TX16	[6]
**orf2010 (fms14)**	GTAGCGAAGAAAATGAGATGG<br>TAACTTGACTGAATCGGTGC	1021	55	TX16	[6]
**orf2514 (fms15)**	AGTTCCAGTTGCGAGTCAGA<br>ATGTAGTCGGATTCGGTGC	989	55	TX16	[6]
***vanB* consensus**	CAAAGCTCCGCAGCTTGCATG<br>TGCATCCAAGCACCCGATATAC	484	58	C68	[1]
***$vanX_B$*-ORFC**	ATCAAGGACTCAACCGTAATT<br>TGAGTTGTGGAAGTCGATTAGAG	873	60	C68	[8]
axe-txe	CTTTAATGGCTCAGGTTTTCCTAA<br>ATGAGGATGCTGAAACACTTATT	351	55	U37	[9]
$rep_{17/pRUM}$	TACTAACTGTTGGTAATTCGTTAAAT<br>ATCAAGGACTCAACCGTAATT	604	52	U37 [10]	[11]
rep_{pLG1}	TTTAAGGCGGATAGAGTTTACAACG<br>CTGATAGGCTTTTAACAGTGTCGTGT	864	56	TX16	[12]
$rep_{2/pRE25}$	GAGAACCATCAAGGCGAAAT<br>ACCAGAATAAGCACTACGTACAATCT	630	56	RE25 [13]	[11]
**ICE*Slu*van Q8**	CAAGTGGTAACGCAGGATGA<br>AAAGATAGCCGTCTGCGTGT	2512/5100	55	5-F9	[14]

References

1. Dahl KH, Simonsen GS, Olsvik Ø, Sundsfjord A (1999) Heterogeneity in the *vanB* gene cluster of genomically diverse clinical strains of vancomycin-resistant enterococci. Antimicrob Agents Chemother 43: 1105-1110.
2. Nallapareddy SR, Weinstock GM, Murray BE (2003) Clinical isolates of *Enterococcus faecium* exhibit strain-specific collagen binding mediated by Acm, a new member of the MSCRAMM family. Mol Microbiol 47: 1733-1747.
3. Bjørkeng EK, Rasmussen G, Sundsfjord A, Sjöberg L, Hegstad K, et al. (2011) Clustering of polyclonal VanB-type vancomycin-resistant *Enterococcus faecium* in a low-endemic area was associated with CC17-genogroup strains harbouring transferable *vanB2*-Tn*5382* and pRUM-like *repA* containing plasmids with *axe-txe* plasmid addiction systems. APMIS 119: 247-258.
4. Leavis H, Top J, Shankar N, Borgen K, Bonten M, et al. (2004) A novel putative enterococcal pathogenicity island linked to the *esp* virulence gene of *Enterococcus faecium* and associated with epidemicity. J Bacteriol 186: 672-682.
5. Rice LB, Carias L, Rudin S, Vael C, Goossens H, et al. (2003) A potential virulence gene, *hylEfm*, predominates in *Enterococcus faecium* of clinical origin. J Infect Dis 187: 508-512.
6. Hendrickx AP, van Wamel WJ, Posthuma G, Bonten MJ, Willems RJ (2007) Five genes encoding surface-exposed LPXTG proteins are enriched in hospital-adapted *Enterococcus faecium* clonal complex 17 isolates. J Bacteriol 189: 8321-8332.
7. Arduino RC, Murray BE, Rakita RM (1994) Roles of antibodies and complement in phagocytic killing of enterococci. Infect Immun 62: 987-993.
8. Dahl KH, Lundblad EW, Røkenes TP, Olsvik Ø, Sundsfjord A (2000) Genetic linkage of the *vanB2* gene cluster to Tn*5382* in vancomycin-resistant enterococci and characterization of two novel insertion sequences. Microbiol 146: 1469-1479.
9. Rosvoll TC, Pedersen T, Sletvold H, Johnsen PJ, Sollid JE, et al. (2010) PCR-based plasmid typing in *Enterococcus faecium* strains reveals widely distributed pRE25-, pRUM-, pIP501- and pHTbeta-related replicons associated with glycopeptide resistance and stabilizing toxin-antitoxin systems. FEMS Immunol Med Microbiol 58: 254-268.
10. Rice LB, Carias LL, Donskey CL, Rudin SD (1998) Transferable, plasmid-mediated *vanB*-type glycopeptide resistance in *Enterococcus faecium*. AntimicrobAgents Chemother 42: 963-964.
11. Jensen LB, Garcia-Migura L, Valenzuela AJ, Lohr M, Hasman H, et al. (2010) A classification system for plasmids from enterococci and other Gram-positive bacteria. J Microbiol Methods 80: 25-43.
12. Rosvoll TC, Lindstad BL, Lunde TM, Hegstad K, Aasnæs B, et al. (2012) Increased high-level gentamicin resistance in invasive *Enterococcus faecium* is associated with *aac(6')Ie-aph(2'')Ia*-encoding transferable megaplasmids hosted by major hospital-adapted lineages. FEMS Immunol Med Microbiol 66: 166-176.
13. Schwarz FV, Perreten V, Teuber M (2001) Sequence of the 50-kb conjugative multiresistance plasmid pRE25 from *Enterococcus faecalis* RE25. Plasmid 46: 170-187.
14. Bjørkeng EK, Hjerde E, Pedersen T, Sundsfjord A, Hegstad K (2013) ICE*Slu*van; a 94-kb mosaic integrative conjugative element conferring interspecies transfer of VanB-type glycopeptide resistance, a novel bacitracin resistance locus and a toxin-antitoxin stabilisation system. J Bacteriol 195: 5381-5390.
