# Supplementary material for: A Multicentre Hospital Outbreak in Sweden Caused by Introduction of a vanB2 Transposon into a Stably Maintained pRUM-Plasmid in an Enterococcus faecium ST192 Clone
Source: PLoS One. 2014 Aug 25;9(8):e103274. doi: 10.1371/journal.pone.0103274 (PMC4143159; doi:10.1371/journal.pone.0103274)
Supplement: Table S2 — Plasmid replication, resistance, toxin-antitoxin system and conjugative transposon genes found in the WGSs of the pre-outbreak isolate VRE576 and the three outbreak isolates VSE1036, VRE1044 and VRE1261. Gene identity refers to the reference sequence. (PDF) [file pone.0103274.s008.pdf]

Table S2. Plasmid replication, resistance, toxin-antitoxin system and conjugative transposon genes found in the WGSs of the pre-outbreak isolate VRE576 and the three outbreak isolates VSE1036, VRE1044 and VRE1261. Gene identity refers to the reference sequence.

Category	Gene	Rep group family	Isolate	Contig	% nt identity	Alignment lengt nt	Reference data		
							**GenBank Acc. No.**	**Locus**	**Species**
Replication genes	CDS1 pRE25	2	VRE0576	contig00101	100.00	1434	X92945	33-1526	*E. faecium*
			VSE1036	contig00084	100.00	1434			
			VRE1044	contig00079	100.00	1434			
				contig00081	89,76	1358			
			VRE1261	contig00085	100.00	1434			
				contig00098	89,76	1358			
	repA pB82	11	VRE0576	contig00054	83.72	215	AB178871	4157-5101	*E. faecium*
			VSE1036	contig00090	100.00	579			
			VRE1044	contig00087	100.00	579			
			VRE1261	contig00091	100.00	579			
	replication initiation protein pRI1	14	VRE0576				EU327398	3161-4114	*E. faecium*
			VSE1036	contig00113	83.50	976			
			VRE1044	contig00109	83.50	976			
			VRE1261	contig00121	83.50	976			
	Putative *repA* pRUM	17	VRE0576	contig00094	100.00	1041	AF507977	20542-21582	*E. faecium*
			VSE1036	contig00075	97.32	1043			
			VRE1044	contig00070	97.32	1043			
			VRE1261	contig00072	97.32	1043			
	replication-associated protein *repA* pLG1		VRE0576	contig00115	98.6	1041	HM565183	40769-41809	*E. faecium*
			VSE1036	contig00071	99.7	1041			
			VRE1044	contig00086	99.7	1041			
			VRE1261	contig00090	99.7	1041			
	Putative plasmid replication protein pCIZ2	unique	VRE0576	contig00086	99.59	738	NC_008259	5690-6427	*E. faecium*
			VSE1036						
			VRE1044						
			VRE1261						
Resistance genes	vancomycin resistance gene *vanB* (D-alanine:D-lactate ligase) Tn*1549*-like		VRE0576	contig00004	99,71	1029	AY655721	4857-5885	*E. faecium*
			VSE1036						
			VRE1044	contig00036	99,71	1029			
			VRE1261	contig00049	99,71	1029			
	erythromycin resistance transferase		VRE0576	contig00100	100.00	738	AF507977	12938-13675	*E. faecium*
			VSE1036	contig00100	99.46	738			
			VRE1044	contig00095	99.46	738			
			VRE1261	contig00106	99.46	738			
	Tetracycline resistance (*tetM*) Tn*916*		VRE0576				X56353	223-2142	*E. faecalis*
			VSE1036	contig00094	94,62	1357			
			VRE1044	contig00089	94,62	1356			
			VRE1261	contig00096	94,62	1357			
TA-system genes	Antitoxin of *axe-axe* pRUM		VRE0576	contig00161	100.00	270	AF507977	5213-5482	*E. faecium*
			VSE1036	contig00062	100.00	270			
			VRE1044	contig00036	100.00	270			
			VRE1261	contig00049	100.00	270			
	Toxin of *axe-txe* pRUM		VRE0576	contig00161	100.00	258	AF507977	4963-5220	*E. faecium*
			VSE1036	contig00062	100.00	258			
			VRE1044	contig00036	100.00	258			
			VRE1261	contig00049	100.00	258			
Conjugative transposon genes	Integrase Tn*1549*		VRE0576	contig00008	99,92	1194	AF192329	32432-33625	*E. faecalis*
			VSE1036						
			VRE1044	contig00036	99,92	1194			
			VRE1261	contig00049	99,92	1194			
	Excisionase Tn*1549*		VRE0576	contig00008	99	201	AF192329	32148-32348	*E. faecalis*
			VSE1036						
			VRE1044	contig00036	99	201			
			VRE1261	contig00049	99	201			
